# Instant Attraction: Immediate Action-Effect Bindings Occur for Both, Stimulus- and Goal-Driven Actions

**DOI:** 10.3389/fpsyg.2012.00446

**Published:** 2012-10-25

**Authors:** Markus Janczyk, Alexander Heinemann, Roland Pfister

**Affiliations:** ^1^Department of Psychology III, University of Würzburg, RöntgenringWürzburg, Germany

**Keywords:** ideomotor theory, action planning, free-choice, forced-choice, action-effects, binding

## Abstract

Flexible behavior is only possible if contingencies between own actions and following environmental effects are acquired as quickly as possible; and recent findings indeed point toward an immediate formation of action-effect bindings already after a single coupling of an action and its effect. The present study explored whether these short-term bindings occur for both, stimulus- and goal-driven actions (“forced-choice actions” vs. “free-choice actions”). Two experiments confirmed that immediate action-effect bindings are formed for both types of actions and affect upcoming behavior. These findings support the view that action-effect binding is a ubiquitous phenomenon which occurs for any type of action.

## Introduction

Human behavior other than unconditioned reflexes is characterized by enormous flexibility. In many situations, humans decide what to do and when to act to achieve their current goals. Such behavior has been investigated thoroughly by researchers of various disciplines and it has been distinguished on several grounds. For the present purpose, we focus on one specific distinction, i.e., that of stimulus- vs. goal-driven actions, and relate it to the central aspect of the present study: the question of whether or not similar action-effect associations are formed for these different kinds of actions. Here we focus explicitly on short-term associations of actions and effects, and our results suggest that short-term associations are formed for both kinds of actions (see also Herwig and Waszak, 2012, for similar conclusions with a different approach).

### Stimulus- and goal-driven actions

In the following, we will distinguish actions by their more or less apparent cause. On the one hand, behavior can be exhibited as a response to environmental demands, such as when hitting the brake pedal upon the perception of a red traffic light. On the other hand, humans often act simply when they decide to do so, i.e., they exhibit instrumental behavior to pursue a self-determined goal. Importantly, this behavior may even be initiated in the absence of any explicit external stimulus demanding for it. Throughout this paper, we refer to these types of actions as “stimulus-” vs. “goal-driven,” respectively^1^.

This distinction highlights the criterion that determines whether an action was executed correctly or not: on the one hand it is the stimulus prompting a specific action; on the other hand it is the goal whose pursuit requires an instrumental action. Furthermore, it should be noted that this dichotomy rarely applies to realistic behavior. Rather, both aspects usually play a role in any given action – yet to a varying degree (see also Passingham et al., 2010). Hence, the dominant aspect must be used to pigeonhole the respective action.

In the laboratory, stimulus-driven actions are typically investigated with forced-choice tasks (Berlyne, 1957): a stimulus appears and entirely determines the appropriate response. Berlyne contrasted this task with free-choice tasks, where a stimulus simply prompts to choose between one of several possible response alternatives. These two types of tasks have been employed widely to investigate stimulus- and goal-driven actions (e.g., Waszak et al., 2005; Keller et al., 2006; Herwig et al., 2007; Pfister et al., 2010, 2011; Wolfensteller and Ruge, 2011), and the present experiments also draw on these methods.

### The role of action-effects in action control

Numerous studies across the last years targeted the role of action-effects in action planning and/or execution. The term of action-effects encompasses any contingent sensory changes that are produced by the action. Regarding the conceptual distinction into stimulus- and goal-driven actions, the role of such action-effects was (and still is) subject to discussion (Herwig et al., 2007; Herwig and Waszak, 2009; Pfister et al., 2010, 2011; Herwig and Horstmann, 2011; Wolfensteller and Ruge, 2011). The theoretical background of this debate is mostly related to ideomotor theory – a general framework of human action control that we summarize in the following (Herbart, 1825; Harleß, 1861; James, 1890/1981; Hommel et al., 2001; for historical papers and reviews, see Stock and Stock, 2004; Shin et al., 2010; Pfister and Janczyk, 2012).

In a nutshell, ideomotor theory assumes that (1) actions are represented by their contingent sensory consequences, i.e., action-effects, and that (2) an action is selected and initiated by mentally anticipating these sensory consequences. These assumptions imply that there are stable and bidirectional associations of actions and their effects. For goal-driven actions, such long-term associations between actions and their contingent effects were demonstrated numerous times in the literature (e.g., Elsner and Hommel, 2001; Hommel et al., 2003; Rieger, 2004; Hoffmann et al., 2009). The respective experiments typically employed two distinct experimental phases. In the *acquisition phase*, participants performed freely chosen actions that were followed by contingent action-effects. For example, participants pressed one of two response keys at their choice and each key press reliably produced a low- or high-pitch tone effect (e.g., left key → low tone, right key → high tone). In the subsequent *test* *phase*, these tones were then presented as stimuli to probe the assumed action-effect association. For example, in forced-choice test phases, participants react to the effects either in an acquisition-compatible (i.e., low tone → left key, high tone → right key) or in the reversed mapping (i.e., high tone → left key, low tone → right key). Such studies consistently found reaction times (RTs) to be faster in the acquisition-compatible mapping than in the reversed mapping (the “non-reversal advantage”), and these results clearly indicate that action-effect associations were built up for the freely chosen actions in the acquisition phase.

As noted above, most studies in this design employed free-choice actions in the acquisition phase (but see Elsner and Hommel, 2004). A systematic comparison of both, free- and forced-choice actions was reported by Herwig et al. (2007). In this study, participants learned action-effect associations for either free- or forced-choice actions. A subsequent forced-choice test phase then probed for resulting action-effect associations. With a free-choice acquisition phase, they replicated the non-reversal advantage of previous studies (e.g., Elsner and Hommel, 2001). In contrast, for the forced-choice acquisition phase, this effect was absent (and in some conditions even numerically reversed). This finding was later shown not to depend on attentional factors (Herwig and Waszak, 2009) and to occur also for eye-movements as response modality (Herwig and Horstmann, 2011).

These findings were taken to suggest that action-effect associations are not built up for stimulus-driven actions. This conclusion, however, is at odds with several findings related to ideomotor theory. For instance, slight variations of the design of acquisition and test phase yielded reliable signs for action-effect learning in forced-choice tasks (Hommel, 1996; Elsner and Hommel, 2004; Pfister et al., 2011), already after very few pairings of actions and effects (Wolfensteller and Ruge, 2011). Furthermore (arbitrary) action-effects were shown to have a pronounced impact in a huge variety of entirely forced-choice tasks (e.g., Hommel, 1993; Ziessler, 1998; Kunde, 2001, 2003; Koch and Kunde, 2002; Rieger, 2007; Janczyk et al., 2009, 2012a; Hubbard et al., 2011).

### The present approach: Short-term action-effect associations

In sum, the evidence whether or not associations of actions and their effects are acquired for stimulus-driven actions is somewhat mixed, yet with a trend toward a positive answer. So far, however, we have only dealt with long-term associations. On a shorter time-scale, features of a particular action (e.g., stimulus, response, and effect) are assumed to be bound into an event-file (Hommel, 1998; Hommel et al., 2001). Although there is evidence that such short-term associations are not necessarily the same as, or a precondition for long-term associations (Colzato et al., 2006), it is still important to know whether a putative difference between stimulus- and goal-driven actions is present in the short-term domain.

In the first study on such short-term action-effect associations, Dutzi and Hommel (2009) reasoned that a sufficiently co-activated (free-choice) response and its contingent effect should be integrated readily into an event-file (Hommel, 1998; Hommel et al., 2001). Encountering the effect again after a short period of time should thus prime the associated response (Hommel, 2007). This *response-repetition bias* was indeed found in four experiments. Thus, action-effects seem to be bound into event-files instantaneously (Dutzi and Hommel, 2009; see also Pfister et al., 2012, for the integration of effects that have been associated with a response on a long-term time scale). Additional evidence from a different paradigm suggests that stimuli occurring after a forced-choice response are similarly bound to the responses (Hommel, 2005, Experiment 2). As there are no direct comparisons in this context, it is unclear whether short-term associations occur similarly for both types of actions. To this end we (1) replicate earlier findings for free-choice responses (Dutzi and Hommel, 2009) and (2) show similar associations for forced-choice actions.

## Experiment 1

Participants performed a task in which each trial consisted of two stages. A first response produced one of two auditory action-effects in a non-predictable manner. Importantly, this response was either a free-choice action (Experiment 1a, replicating the paradigm of Dutzi and Hommel, 2009) or a forced-choice action (Experiment 1b). Shortly thereafter, the same or the other tone was presented, prompting a free-choice response. For the free-choice actions of Experiment 1a, we expected to replicate the response-repetition bias when the effect tone was repeated. If the same binding mechanism operates for forced-choice actions, a similar bias should be observed in Experiment 1b. In contrast, this bias should be absent if action-effect binding does not take place under these circumstances.

### Method

#### Participants

Seventeen participants performed in Experiment 1a (mean age = 27.8 years, 12 female), and another 16 participants performed in Experiment 1b (mean age = 21.6 years, 13 females). Participants were undergraduate students from the University of Würzburg and were naïve regarding the hypotheses underlying this experiment.

#### Apparatus and stimuli

Visual stimuli were presented in white against a black background. The imperative stimulus in the first stage of each trial was a string of 13 centrally presented asterisks in Experiment 1a (see Dutzi and Hommel, 2009) and a small white square presented below and to the left or right of a fixation cross in Experiment 1b (see Herwig et al., 2007; Wolfensteller and Ruge, 2011). Tones were 50 ms sinusoidal tones of either 300 or 900 Hz presented via headphones. Responses were given via the left and right control key of a standard computer keyboard using the index-finger of the left or right hand.

#### Procedure

**Figure 1:**
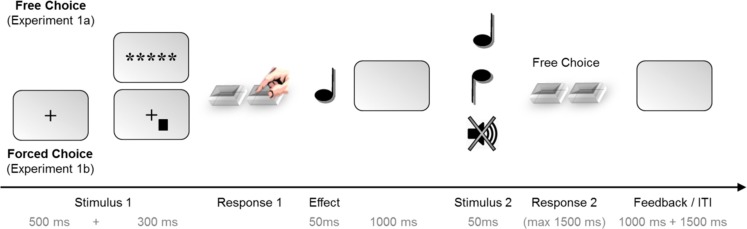
**Trial structure for Experiments 1a and 1b, where the task (free-choice vs. forced-choice) was manipulated between-subjects**. Each trial consisted of two stages: first, a free- or forced-choice response randomly produced one of two possible effect tones. Then, a second tone appeared and prompted the participants to choose a second response; this tone was either the previous effect tone or the alternative tone. In 25% of the trials, no tone appeared as Stimulus 2, indicating a no-go trial. Experiment 2 employed the same trial structure but the task (free- vs. forced-choice as Response 1) was now varied within-subjects.

The trial procedure is illustrated in Figure 1. In Experiment 1a, each trial began with the presentation of the asterisks for 300 ms (Stimulus 1). Participants were to freely choose from both responses at leisure (Response 1). This key press triggered one of the two tones at random (Effect). After 1000 ms, either a second tone occurred (Stimulus 2; go trials) or not (no-go trials). In go trials, the second tone was either the previous effect tone or the alternative tone. Participants were then to freely choose one of the response keys within a time window of 1500 ms. Following late responses or responses in no-go trials, visual error feedback was provided for 1000 ms, and the next trial started after an inter-trial interval (ITI) of 3000 ms. The trial procedure of Experiment 1b was identical except for the presentation of Stimulus 1. Here, each trial started with a fixation cross for 500 ms. Then, Stimulus 1 was presented to the left or right of fixation (300 ms) prompting a speeded response with the corresponding key. Wrong responses to Stimulus 1 prompted an error feedback (1000 ms) and the trial was canceled afterward. For correct responses, the trial continued just as in Experiment 1a.

Participants completed three experimental blocks with 64 trials each. Of these trials, 16 trials were no-go trials. In 24 trials the effect tone was repeated as Stimulus 2 (congruent go trials), and in the remaining 24 trials the other tone was played as Stimulus 2 (incongruent go trials). Ten practice trials were completed prior to the experimental blocks. Participants were tested individually in a single session of about 20 min, and they received written instructions prior to the experiment. For free-choices, participants were instructed to decide on the response as spontaneously as possible and not to pursue any specific strategies. Furthermore, they were encouraged to produce both response alternatives about equally often throughout the experiment.

### Results

**Figure 2:**
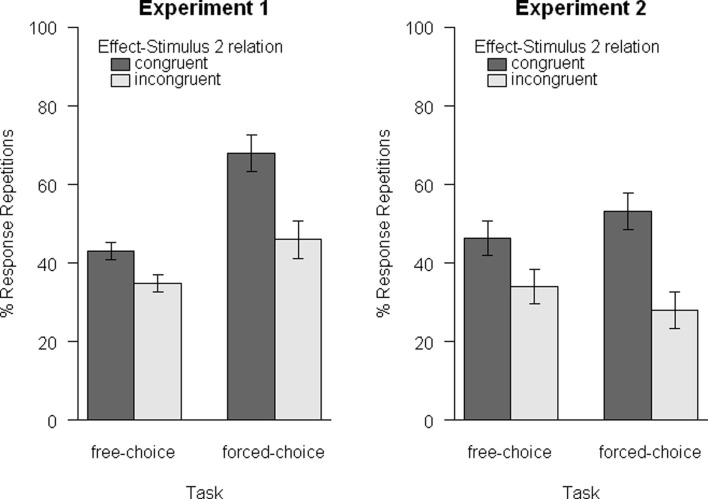
**Mean response-repetition rates as a function of task (free-choice vs. forced-choice) and relation of Effect and Stimulus 2 (congruent vs. incongruent)**. A repetition bias for congruent trials emerged consistently for both tasks in both experiments. Error bars are within-subjects standard errors, calculated separately for each comparison of congruent and incongruent trials (Loftus and Masson, 1994).

The main dependent variable was the proportion of go trials with response-repetitions (*repetition rate*; see Figure 2, left panel)^2^. For Experiment 1a, we excluded go trials with late responses (0.9%) and for Experiment 1b we excluded trials with wrong responses to Stimulus 1 (1.0%) and go trials with late responses (0.8%). We then compared the repetition rates for congruent and incongruent go trials with separate one-tailed *t*-tests. Erroneous responses in no-go trials were given in 3.9 and 2.1% of the trials in the free- and forced-choice task, respectively.

For *Experiment 1a (free-choice)*, we excluded two participants from the analysis because they had chosen only a single key as Response 1 in 91.6 and 99.5% of the trials, respectively. The remaining 15 participants chose both keys about equally often for Response 1 [48.8 vs. 51.2%, χ^2^(1) = 1.61, *p* = 0.205].

The results are visualized in Figure 2 (left panel). Crucially, the repetition rate was significantly higher in congruent as compared to incongruent trials, *t*(14) = 2.58, *p* = 0.011, *d* = 0.94. The same results emerged for *Experiment 1b (forced-choice)*, *t*(15) = 3.29, *p* = 0.002, *d* = 1.16.

To test for differences between free- and forced-choice actions, we performed an additional *between experiment analysis* by means of a 2 × 2 ANOVA with trial-type (congruent vs. incongruent) as a within-subjects factor and experiment (1a vs. 1b) as a between-subjects factor. This analysis yielded a significant main effect of trial-type, *F*(1, 29) = 15.82, *p* < 0.001, $\eta_{p}^{2}$ = 0.35, confirming the higher repetition rate for congruent as compared to incongruent trials. Secondly, a significant main effect of experiment, *F*(1, 29) = 6.52, *p* = 0.016, $\eta_{p}^{2}$ = 0.18, indicated a generally higher repetition rate in Experiment 1b. Finally, the interaction of both factors approached significance, *F*(1, 29) = 3.48, *p* = 0.072, $\eta_{p}^{2}$ = 0.11, suggesting a larger effect of trial-type in Experiment 1b than in Experiment 1a.

### Discussion

The purpose of Experiment 1 was to replicate and extend previous findings of immediate action-effect binding and its impact on subsequent free response choices (Dutzi and Hommel, 2009). To this end, a free-choice response (Experiment 1a) or a forced-choice response (Experiment 1b) produced an auditory action-effect. Shortly after this action-effect, a second tone prompted a free-choice response. This tone was either the previous action-effect or another tone. As predicted, tone repetitions biased participants to repeat the previous response. For Experiment 1a (free-choice), mean repetition rates were in the range reported earlier by Dutzi and Hommel (2009). For Experiment 1b (forced-choice), overall repetition rates were higher and – at least numerically – the bias was even larger than in Experiment 1a. Therefore, and because of the between-subject manipulation in Experiment 1, we conducted Experiment 2 where both, free- and forced-choice responses were implemented within-subjects.

## Experiment 2

In Experiment 1, we replicated the response-repetition bias reported by Dutzi and Hommel (2009), when participants performed their first response in a free-choice task. More importantly, we found the same pattern when this response resulted from a forced-choice situation. This indicates that short-term action-effect binding takes also place in this situation and is not restricted to free-choice actions. Somewhat unexpected, this bias was numerically even larger for the forced- compared to the free-choice situation. In Experiment 2 we strived to replicate this finding using a within-subjects design.

### Method

Twelve new participants from the city of Würzburg (mean age = 25.0 years; 8 females) performed in this experiment. Procedural details were as in Experiment 1 with one exception: all participants performed in both, the free- and the forced-choice variant. Accordingly, task (free-choice vs. forced-choice) was introduced as a second repeated measure. The order of these tasks was counterbalanced across participants.

### Results

In the free-choice part, 1.0% of the go trials were excluded because of late responses. In the forced-choice part, trials with errors in response to Stimulus 1 were excluded (1.8%) as well as late responses to Stimulus 2 (2.0%). Participants chose both keys about equally often for Response 1 in the free-choice task [each 50.0%, χ^2^(1) < 0.01, *p* = 0.967]. Mean response-repetitions in correct trials were submitted to an ANOVA with trial-type (congruent vs. incongruent) and task (free-choice vs. forced-choice) as repeated measures. Results are illustrated in Figure 2 (right panel). Erroneous responses in no-go trials were given in 5.6 and 2.3% of the trials in the free- and forced-choice task, respectively.

Response-repetitions were again significantly more likely for congruent than for incongruent trials, *F*(1, 11) = 10.15, *p* = 0.009, $\eta_{p}^{2}$ = 0.48. Importantly, this main effect was qualified by the significant interaction of trial-type and task, *F*(1, 11) = 8.72, *p* = 0.013, $\eta_{p}^{2}$ = 0.44. The main effect of task was not significant, *F*(1, 11) = 0.02, *p* = 0.880, $\eta_{p}^{2}$ < 0.01. One-tailed *t*-tests showed significantly more repetitions in congruent trials for both, the free-choice task, *t*(11) = 1.99, *p* = 0.036, *d* = 0.81, and the forced-choice task, *t*(11) = 3.92, *p* = 0.001, *d* = 1.60. Additionally, the size of these biases was strongly correlated across participants, *r*(12) = 0.75, *p* = 0.005.

### Discussion

The results of Experiment 2 replicated the pattern already observed in Experiment 1. A reliable response-repetition bias emerged for congruent trials for both tasks, and was again larger in the forced-choice task than in the free-choice task. Thus, again short-term action-effect associations were observed for both, free- and forced-choice actions. The positive correlation of the biases also indicates that the associations are built up to a similar degree not only on the group level, but also on the level of the individual.

## General Discussion

In the present study we investigated whether stimulus- and goal-driven actions differ with regard to short-term action-effect associations. In general, there is good evidence that stimuli and their corresponding responses are integrated into event-files (Hommel, 1998; Hommel et al., 2001). Similar integrations have previously been shown for the effects of goal-driven (free-choice) actions (Dutzi and Hommel, 2009), but only indirectly for stimulus-driven actions (Hommel, 2005, Experiment 2).

In the two present experiments, participants performed one of two manual responses, either as determined by an imperative stimulus (forced-choice task; stimulus-driven actions) or freely chosen by the participant (free-choice task; goal-driven actions). Each response randomly triggered one of two tones as an action-effect. Briefly thereafter, the same, the other, or no tone was played. In case of a second tone, participants freely chose between both responses again. Participants tended to repeat the first response more often when the effect tone was repeated to signal the (second) response than when it was not. Thus, short-term action-effect associations, i.e., the integration of actions and their effects into an event-file (Hommel, 1998), were evident in our experiments. Crucially, this was true for both, goal-driven, free-choice actions (Dutzi and Hommel, 2009) and stimulus-driven, forced-choice actions. Indeed, the observed bias was even larger for the stimulus-based actions.

### Short-term and long-term action-effect associations

These results suggest that actions and their effects are integrated regardless of whether the action is classified as stimulus- or goal-driven. Herwig and Waszak (2012) tackled a similar question with a slightly different experimental approach. They also employed two responses in each trial with the first response being either free- or forced-choice. Again, this response produced an action-effect and a second stimulus prompted the second response. This second stimulus could share features with the previous effect or not. In contrast to the present setup, however, the second response was forced-choice throughout. Accordingly, RTs and percentage errors were analyzed instead of repetition rates. Still, their results mostly converge with the present findings. However, in contrast to the present results, Herwig and Waszak did not find any hints toward a larger bias for forced-choice actions and they accordingly conclude that short-term binding results equally for free- as well as forced-choice actions.

In light of these differences, we are cautious about drawing definite conclusions from the observed differences between the present free- and forced-choice tasks in terms of stronger binding for forced-choice actions. Instead, passing control to the environment in a forced-choice task might simply have rendered the participants more susceptible for other environmental influences such as response tendencies invoked by the perception of previous action-effects. Thus, the more pronounced effects for the present forced-choice tasks need not necessarily imply a genuinely stronger binding. Additionally, this case required continuous task switches from forced- to free-choices and back. This may have increased attention to task-related stimuli and might have led to a stronger effect in the forced-choice condition, too. Regardless of this interesting difference, these results converge in the notion that short-term action-effect associations are built for both, stimulus- and goal-driven actions. Thus, on this level there is little reason for the assumption of profound and qualitative differences between these actions.

However, our data do not speak toward differences in long-term associations. Colzato et al. (2006) preferred the interpretation that short-term and long-term associations are not necessarily dependent on each other, although the existence of stable long-term memory representations appears to affect the time-course of short-term associations over a series of trials. Herwig and Waszak, 2012, Experiment 3) complemented their approach by testing additionally for long-term associations that may have evolved from an acquisition phase, where their participants showed reliable short-term associations. In line with their previous findings (Herwig et al., 2007; Herwig and Waszak, 2009; Herwig and Horstmann, 2011), a reliable long-term association of actions and corresponding effects was only found for those participants who performed the free-choice version of their acquisition task. Herwig and Waszak (2012) suggest that redundant information might be represented less and less strongly during repeated occurrences, and eventually is thus not integrated into long-term associations. Thus, in the case of forced-choice actions, action-effects are more and more identified as redundant; consequently their representation should diminish and eventually do not leave a long-term trace. Herwig and Waszak also suggest that such additional assumptions can explain the results of Wolfensteller and Ruge (2011). The latter authors observed reliable “long-term” associations after only a few forced-choice acquisition trials. According to Herwig’s and Waszak’s reasoning then, the brevity of the acquisition phases simply may not have allowed to firmly identifying the redundancy of the effects. As a consequence, they were still well represented and integrated with the responses – as became evident in their test phases.

Nevertheless, this hypothesis cannot explain the whole range of findings. It is difficult to see why free-choice test phases reliably reveal long-term action-effect associations, even when acquired during a long acquisition phase (Kühn et al., 2009, Experiment 3; Pfister et al., 2011). Furthermore, it cannot explain why slight variations of the experimental design (e.g., including more response alternatives) do yield reliable effects also for purely forced-choice settings (Hommel, 1996; Elsner and Hommel, 2004).

A different perspective that might account for these findings is based on the *intentional weighting* of feature codes (Hommel, 1996). According to this view, any feature code that covaries with the response is represented automatically. Intentional control over the available codes is possible by assigning different weights to the represented features. This account is in line with a variety of findings relating to perception and action (Memelink and Hommel, in press). The absence of experimental evidence for action-effect associations in forced-choice test phases following prolonged forced-choice acquisition phases (Herwig et al., 2007; Herwig and Waszak, 2009) might thus indicate that small weights are assigned to the represented action-effects even though an action-effect association does exist. This process might be supported by the fact that action-effects were explicitly rendered task-irrelevant in these studies.

In this view, free-choice (i.e., goal-driven) actions simply increase the tendency to use action-effects for action control by assigning a stronger weight to the relevant codes (Pfister et al., 2010, 2011). Furthermore, free response choices are not the only way to increase the weight that is assigned to action-effects. Other relevant factors might be instructions (Hommel, 1993), task-relevance (Ansorge, 2002), or saliency of action-effects that are relevant for the task at hand (e.g., Kunde et al., 2007, 2012; Janczyk et al., 2012a,b). Most importantly, however, this account does not assume qualitative differences between stimulus- and goal-driven actions regarding the underlying learning mechanisms. This implication is further supported by the present results which suggest similar mechanisms to mediate short-term bindings for both, stimulus- and goal-driven actions.

## Conclusion

The present study investigated short-term associations between actions and their following effects. More precisely, we addressed the formation of such short-term bindings for stimulus-driven (forced-choice) and goal-driven (free-choice) actions. Results indicate that ensuing action-effects are readily associated to both types of actions. On a broader scale, these findings are also in line with the common definition of “actions” for which “goal-directedness” is a key element, independent of the more or less apparent cause/motivation of an action.

## Conflict of Interest Statement

The authors declare that the research was conducted in the absence of any commercial or financial relationships that could be construed as a potential conflict of interest.

## Footnotes

^1^These types of behavior have, among others, been termed “stimulus-based” or “externally triggered” vs. “intention-based” or “self-initiated,” respectively (e.g., Waszak et al., 2005; Herwig et al., 2007; Brass and Haggard, 2008; Passingham et al., 2010). However, if people show a particular behavior in response to a given stimulus (i.e., “stimulus-based”), the relevant association between stimulus and behavior has to be established beforehand. This, in turn requires intention as well. Exner (1879; cited in Hommel, 2000) described this kind of behavior as the “prepared reflex.” Thus, stimulus-based actions clearly require intentions as well, at least when setting up the association between stimulus and response. We therefore use the labels stimulus- vs. goal-*driven* throughout this article.

^2^One could in principle also analyze RTs as the dependent variable. However, it is rather difficult to interpret any pattern that might emerge from such an analysis, rendering RTs an inappropriate measure in this context, as has been argued in detail by Dutzi and Hommel, 2009, Footnote 1). Exploratory analyses of the present data yielded no systematic pattern across both experiments. Descriptive RTs to Stimulus 2 are summarized in the Appendix.

## Appendix

**Table A1:** **Mean RTs (ms) to Stimulus 2 from Experiments 1 and 2 as a function of Effect – Stimulus 2 relation**.

Effect – S2 relation	Experiment 1		Experiment 2	
	Free-choice	Forced-choice	Free-choice	Forced-choice
Congruent	530	530	525	557
Incongruent	512	525	493	568

*Note that task (free- vs. forced-choice) was varied between-subjects in Experiment 1 and thus refers to Experiment 1a and 1b, respectively. RTs are based on correct trials only and RTs deviating from the respective cell mean by more than 3SDs were considered outliers*.
